# Bicarbonate and dichloroacetate: Evaluating pH altering therapies in a mouse model for metastatic breast cancer

**DOI:** 10.1186/1471-2407-11-235

**Published:** 2011-06-10

**Authors:** Ian F Robey, Natasha K Martin

**Affiliations:** 1 Arizona Respiratory Center, University of Arizona, Tucson Arizona, USA; 2 Centre for Mathematical Biology, Mathematical Institute, Oxford University, 24-29 St Giles', Oxford, OX1 3LB, UK; 3 Department of Social Medicine, University of Bristol, Canynge Hall, 29 Whatley Road, Bristol, BS8 2PS, UK

**Keywords:** Tumor, pH, Acidity, Dichloroacetate, Sodium bicarbonate

## Abstract

**Background:**

The glycolytic nature of malignant tumors contributes to high levels of extracellular acidity in the tumor microenvironment. Tumor acidity is a driving force in invasion and metastases. Recently, it has been shown that buffering of extracellular acidity through systemic administration of oral bicarbonate can inhibit the spread of metastases in a mouse model for metastatic breast cancer. While these findings are compelling, recent assessments into the use of oral bicarbonate as a cancer intervention reveal limitations.

**Methods:**

We posited that safety and efficacy of bicarbonate could be enhanced by dichloroacetate (DCA), a drug that selectively targets tumor cells and reduces extracellular acidity through inhibition of glycolysis. Using our mouse model for metastatic breast cancer (MDA-MB-231), we designed an interventional survival study where tumor bearing mice received bicarbonate, DCA, or DCA-bicarbonate (DB) therapies chronically.

**Results:**

Dichloroacetate alone or in combination with bicarbonate did not increase systemic alkalosis in mice. Survival was longest in mice administered bicarbonate-based therapies. Primary tumor re-occurrence after surgeries is associated with survival rates. Although DB therapy did not significantly enhance oral bicarbonate, we did observe reduced pulmonary lesion diameters in this cohort. The DCA monotherapy was not effective in reducing tumor size or metastases or improving survival time. We provide *in vitro *evidence to suggest this outcome may be a function of hypoxia in the tumor microenvironment.

**Conclusions:**

DB combination therapy did not appear to enhance the effect of chronic oral bicarbonate. The anti-tumor effect of DCA may be dependent on the cancer model. Our studies suggest DCA efficacy is unpredictable as a cancer therapy and further studies are necessary to determine the role of this agent in the tumor microenvironment.

## Background

The extracellular pH of malignant tumors is acidic (pH 6.5-6.9) compared to normal tissue (pH 7.2-7.4) [1-3]. Tumor acidity is thought to play a critical role in chemoresistance [4-6] and promotion of metastatic potential. For example, acidic culture conditions increase filopodia formations and expression of proteolytic enzymes involved in invasion [7]. Other reports have described enhanced invasiveness [8] and increased cathepsin B activity in acid-cultured tumor cells [9]. Acid-mediated metastatic potential has been demonstrated *in vivo *as well. Tumor cells pre-treated under acidic pH prior to tail vein injection formed a greater number of pulmonary metastases as well upregulated activity of metastatic effectors such as serine proteases and angiogenic factors [10].

Although decreased extracellular tumor pH is strongly associated with numerous cellular mechanisms including carbonic anhydrases [11], vacuolar ATPases [12], and sodium-ion exchangers [13], glycolysis is considered the major factor in promoting tumor acidity. Excessive tumor glycolysis, even in the presence of oxygen, is a hallmark of malignancy and leads to increased production of lactic acid [14,15]. The notion that acidic pH is sufficient for driving tumor cell invasion inspires the corollary prediction that neutralizing acidic tumor pH inhibits invasion and slows the spread of metastases. This question was tested in a survival study of tumor bearing mice. Female severe combined immunodeficient (SCID) mice with MDA-MB-231 breast tumor xenografts were chronically administered drinking water with sodium bicarbonate. This study reported that systemic bicarbonate buffered the extracellular pH in tumors to neutral levels (a pH of 7.2) and inhibited the spread of metastases which led to improved survival. Systemic bicarbonate was also effective against the spread of metastases in a model for prostate cancer [16].

Certain determinations are required before considering systemic buffering of tumor acidity as feasible approach to cancer treatment. Effective doses, susceptible tumor types, ideal buffers, safety approaches, and other factors in the tumor microenvironment would need to be identified and validated. To assist our understanding of these phenomena, mathematical models were developed to predict the safety and efficacy of systemic buffering of tumor acidity in humans. Our findings suggest that chronic use of oral bicarbonate as a cancer intervention is limited. Safe doses for consumption limit the amount of buffering to counteract tumor cell production of extracellular protons. In a previous study, it was reported that the saturating dose of oral bicarbonate in mice (which roughly translates to about 0.18 g/kg/day in humans) was only sufficient to counteract the acid load of a 15 mg tumor consisting of about 100,000 cells or 1.0 mm^3 ^[16]. Moreover, chronic application of oral bicarbonate at doses higher than 0.5 g/kg/day is predicted to induce systemic alkalosis (Martin N, Robey I, Gaffney E, Gillies R, Gatenby R, Maini P: Predicting the Safety and Efficacy of Buffer Therapy to Raise Tumor pHe: An Integrative Modeling Study, submitted). Therefore, it is thought that safety and efficacy of oral bicarbonate could be enhanced by other reagents that directly or indirectly target the processes driving extracellular tumor acidity.

One possible method to enhance extracellular pH buffering is the orphan drug dichloroacetate (DCA). This analogue of acetic acid has been shown to selectively target tumor cell metabolism by indirectly activating pyruvate dehydrogenase (PDH) through inhibition of pyruvate dehydrogenase kinase (PDK) activity. Upregulated PDH activity shifts metabolism of pyruvate to lactate under the glycolytic cascade towards production of acetyl-CoA, which is fed into the Krebs Cycle to drive oxidative phosphorylation. This shift results in decreased cellular glycolysis with a subsequent increase in extracellular pH. It also triggers multiple apoptotic signaling events in the mitochondria [17,18]. To explore the question of whether DCA and bicarbonate is a viable therapeutic intervention in metastatic disease we conducted an interventional survival study in mice bearing MDA-MB-231 metastatic breast tumor xenografts divided into four treatment groups 1) untreated; 2) bicarbonate; 3) DCA and 4) DCA-bicarbonate (DB).

## Methods

### Animal experiments

All animals were maintained under Institutional Animal Care and Use Committee (IACUC) - approved protocols at the University of Arizona. Six to eight-week-old female SCID mice received orthotopic injections of 5 × 10^6 ^MDA-MB-231/eGFP tumor cells in a mammary fatpad. Tumor bearing animals were randomized into 1) untreated; 2) sodium bicarbonate (Sigma, St. Louis, MO), 200 mM (equivalent to 3.2 g/kg/day in mice); 3) sodium dichloroacetate (DCA) (Sigma, St. Louis, MO), 5 mM (equivalent to 112 mg/kg/day); and 4) sodium bicarbonate (200 mM) plus sodium dichloroacetate (5 mM) combination (DB) water six days following tumor inoculation. Starting cohort sizes were 16 per group. Animals were monitored and maintained by the Experimental Mouse Shared Services (EMSS) core facility of the Arizona Cancer Center, Tucson Arizona. Volumes of primary tumors in mammary fat pads were measured twice weekly and calculated from orthogonal measurements of external dimensions as (width)^2 ^× (length)/2. Surgical resections of primary tumors occurred between days 28-50 when tumors were approximately 500 mm^3^. Mice were euthanized when tumor burden was excessive (primary, intraperitoneal, or lymph node > 2000 mm^3^) or when mice progressed to a moribund state. The EMSS technician, who was blind to the study, monitored the mice twice weekly. Notification to euthanize was received by the investigator on the day of a monitoring visit and study termination endpoints did not exceed 48 hours. Animals were euthanized by cervical dislocation.

Urine was obtained by applying gentle abdominal pressure against the mouse for 10 sec over plastic film. Urine was collected by micropipette and transferred to a 0.6 mL tube for pH measurement. Serum was collected by heart puncture after mice were euthanized. Blood was centrifuged and serum was collect for pH measurement. Urine and serum pH was measured using a Mettler Toledo pH meter with an InLab^® ^Micro probe.

### Metastases measurements

Upon termination of the survival experiment, gross necropsy was used to identify tumor metastases. Green fluorescent tumors were detected by the Illumatool Bright Light System (LT-9500) using a 470 nm/40 nm excitation filter (Lightools Research) and imaged using a mounted PowerShot SD750 digital camera (Canon, Lake Success, NY). The images were captured at the same focal plane in the presence of 480-nm excitation and > 490-nm filtered emission. Whole lung image data were analyzed with Adobe Photoshop 5.0 using the "magic wand" tool to select lung area and green fluorescent tumor lesions. Pixel area of the selected images was measured using ImageJ (http://rsb.info.nih.gov/ij/).

### Cell culture experiments

MDA-MB-231 cells were obtained from the American Type Culture Collection (Rockville, MD) and maintained in Dulbecco's Modified Eagle's Medium/F-12 supplemented with 10% FBS. *Crystal violet assay*: Cells, growing in log phase, were plated in 96-well culture plates at a density of 5 × 10^4 ^cells/mL. The following day cells were exposed to titrating doses of DCA suspended in fresh growth media from 80 mM to 2.5 mM at 20% (normoxia) or 1% O_2 _(hypoxia). Untreated cells in growth media were included as negative controls. After the 24 hour dose period, cells were fixed with 0.025% gluteraldehyde then stained with 0.1% crystal violet. Stained cells were washed with water then resuspended in 10% acetic acid. Absorbance was measured at 590 nm using a Victor3™ plate reader (PerkinElmer, Waltham, MA). *Lactate production measurements*: Cells growing in 24-well plates were washed once with phosphate buffered saline then cultured with 100 μL in 10 mM D-(+)-glucose-supplemented, serum-free RPMI for 1 hour incubation at 37°C in a humidified 5% CO_2 _atmosphere, 20% or 1% O_2_. Lactate levels were quantified using an enzymatically coupled lactic acid detection reagent (Sigma). Ten microliters of lactate supernatant were assayed with 90 μL lactate reagent in 96-well plates. Mixtures were incubated for 5 minutes in the dark at ambient temperature. Absorbance of colorimetric assay was measured at 450 nm. Lactate production rates were calculated from an internal standard curve and expressed as nmol/min/mg protein. Cellular protein was obtained by lysing cells with 100 μL 0.1 N NaOH for 1 minute then neutralizing lysate with an equal volume of 0.1 N HCl. The protein concentration was determined using and internal bovine serum albumin standard curve in a Bradford Assay (Pierce, Rockford, IL). Absorbance of colorimetric assay was measured at 630 nm.

### Statistics

Statistical calculations were determined using the analysis feature in GraphPad Prism version 4.03 for Windows (GraphPad Software, San Diego CA, http://www.graphpad.com). Unpaired, two-tailed t-tests were used to determine if means were significantly different between untreated and treated groups. Categorical analyses were carried out using a two-tailed Fischer's Exact test. A *p*-value of less than 0.05 was considered to be statistically significant. Numerical data values are represented as mean ± SEM.

## Results

### Chronic oral administration of DB has no adverse systemic effects

**Figure 1:**
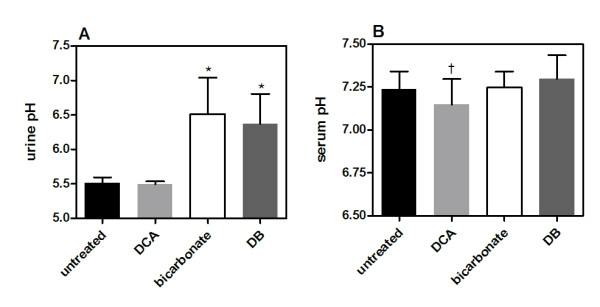
**Comparison of urine and serum pH in treatment groups**. A) Urine samples were collected from tumor bearing mice at eight different time points over a 3 week period from 3-10 mice in each treatment group. At time zero before starting treatment, urine pH in all groups averaged 5.59 ± 0.2 with no statistical difference between groups. The first collection time after starting treatment was at 24 hours. Urine pH measurements from all collection times were averaged. Asterisks (_*_) designate average urine pH values that were statistically significant from untreated mouse group by student's two-tailed t-test (*p *< 0.001). The cross (†) designates average urine pH values between the DCA and DB treated groups were statistically significant (*p *< 0.03). B) Serum pH was measured in euthanized mice. Differences in serum pH between groups were not statistically significant. Error bars are expressed as SEM.

Our previous studies have found that chronic oral bicarbonate significantly increases urine pH in tumor bearing mice by approximately 1.4 fold over a 21 day period. This is a consequence of excess systemic bicarbonate and raises concerns about the safety of long term use in humans (Martin N, Robey I, Gaffney E, Gillies R, Gatenby R, Maini P: Predicting the Safety and Efficacy of Buffer Therapy to Raise Tumor pHe: An Integrative Modeling Study, submitted). Urine pH in DCA treated mice was the same as measured in untreated mice. Urine pH was similar between bicarbonate and DB treated mice (Figure 1A). In the post-survival study there were no statistically significant changes in serum pH between any of the groups. A marginal, but significant serum pH difference (*p *< 0.03) was observed between DB and DCA treated mouse serum (Figure 1B).

### Primary tumor growth was unaffected by treatments

**Figure 2:**
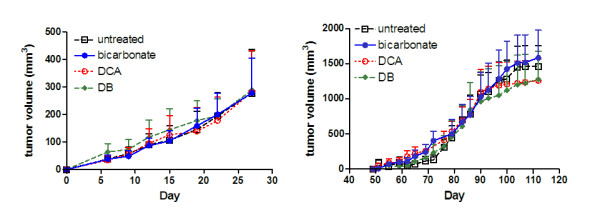
**Effect of bicarbonate, DCA and DB treatments on primary tumor growth**. Growth rates of primary tumors are expressed in mm^3^/time (days). Plots indicate none of the treatments exhibited a measurable effect on primary tumor growth at the beginning of the study (A) or from the time of the tumor resections to study termination (B). Error bars are expressed as SEM. Two-tailed, unpaired t-test, between these groups yielded a *p *> 0.9.

**Figure 3:**
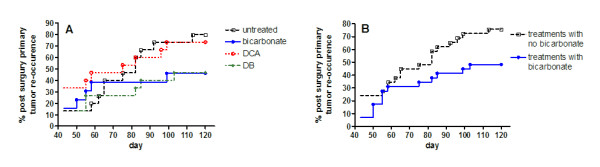
**Primary tumor re-occurrence after survival surgeries**. Survival surgeries occurred between day 28 and 50. Average resection day occurred on day 37 ± 8. Primary tumor sizes in all treatment groups averaged 490 ± 12 mm^3^. Primary tumors started to re-grow around day 50. Primary tumor re-occurrence is plotted over the survival time course as Kaplan-Meier curve. A plot point represents the day when a primary tumor was first measured after surgery. In mice where no primary tumor was observed in post-study necropsy experiments, a value of zero was recorded for day 120. A) Plots for all experimental cohorts (*p *= 0.29). B) Curves comparing groups that were not administered bicarbonate (untreated and DCA) and groups treated with bicarbonate (bicarbonate and DB) (*p *= 0.046).

Previous studies have shown that oral administration of DCA can slow the rate of tumor growth *in vivo *[17]. In our study, treating MDA-MB-231 tumor bearing mice with DCA and DB did not impact primary tumor growth. Tumors in all cohorts grew at an equal rate before they were resected (Figure 2A). The primary tumor growth rates in the bicarbonate and DB treated groups were consistent with earlier findings in bicarbonate treated mice. The results confirm that bicarbonate buffering of tumor acidity does not function to inhibit primary tumor growth [16]. In animals where primary tumors re-occurred, growth rates were not statistically different between groups (Figure 2B). After resection, most primary tumors grew back in the untreated and DCA treated mice. In bicarbonate and DB treated mice, less than half of the tumors grew back after tumor resection (Table 1). Primary tumor reoccurrence was not statistically significant when comparing individual treated cohorts to the untreated group (Figure 3A), but non-bicarbonate treated cohorts (untreated and DCA) had a significantly higher rate of tumor reoccurrence after survival surgery than bicarbonate treated cohorts (bicarbonate and DB) (*p *< 0.05) (Figure 3B)

**Table 1:** Fraction and percentage of mice in each treatment group with re-occurring primary tumors, and with intestinal, mesentery, lymph node, and pulmonary metastatic involvement

	untreated	bicarbonate	DCA	DB
**Re-occurring primary tumors**	12/15 (80%)	6/13 (46.2%)	11/15 (73.3%)	7/15 (46.7%)
**Intestinal**	5/14 (35.7%)	0/11 (0%)	3/14 (21.4%)	2/15 (13.3%)
**Mesentery**	2/14 (14.3%)	0/11 (0%)	2/14 (14.3%)	3/15 (20%)
**Lymph node**	9/14 (64%)	3/11 (27%)	5/14 (35.7%)	7/15 (47%)
**Lung**	11/14 (78.6%)	1/13 (7.7%)	6/13 (46.2%)	10/15 (66.7%)

### Oral administration of bicarbonate and DCA-bicarbonate improves survival over DCA and untreated mice

**Figure 4:**
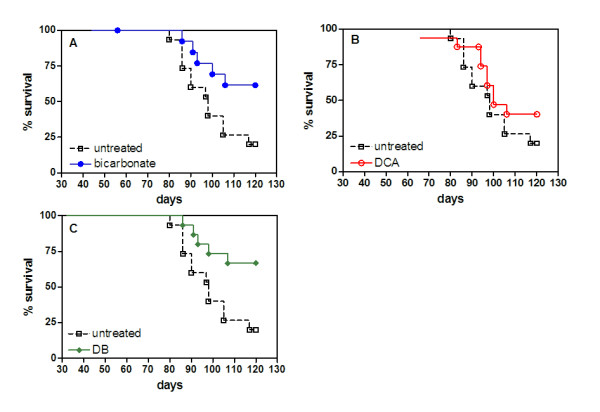
**Effect of bicarbonate, DCA, and DB on and survival**. MDA-MB-231 cells were stably transfected to express neomycin-resistant pcDNA3/EGFP (16). MDA-MB-231/eGFP cells (5 × 10^6^) were injected into inguinal mammary fat pads of animals that were randomized into bicarbonate, DCA, DB, and control groups (n = 16 per group) 6 days post inoculation. Tumors were allowed to grow for 5 to 6 wk (to a volume of approximately 500 mm^3^), at which time they were surgically removed. After survival surgeries, the disease model was allowed to progress until tumor burdens or morbidity criteria warranted the mice to be euthanized. The survival experiment proceeded to day 120. At time of sacrifice, mice were necropsied by examination with a fluorescence dissecting scope. Data from this experiment are plotted as Kaplan-Meier survival curves (A) bicarbonate (*p *= 0.03), (B) DCA (*p *= 0.2), and (C) DB (*p *= 0.01). Treated mice are represented as solid lines compared to the untreated group represented as a dashed line. The difference in the survival curve for the treated versus untreated animals was evaluated by log-rank test.

Because our study design chronically administered DCA over a 17 week period, we limited the drinking water concentration to 5 mM (0.75 g/L) to avoid potential development of hepatic carcinogenesis [19]. Treatments were started 6 days after tumor injections and were continued for the remainder of the study. Mice received survival surgeries when tumors reached approximately 490 mm^3 ^(± 95). On average, these surgeries occurred on day 37 (± 8). Mice that died from surgical procedures were omitted from the study. The mice were maintained on cohort-specific treatment regimens until conditions related to tumor burden required animals to be euthanized. Mice were then analyzed to score and measure tumor metastases. Spontaneous deaths occurred in all groups. Complete postmortem analysis could not be obtained in some of these deaths. These include one in the untreated group, two in the bicarbonate group, two in the DCA group, and one in the DB group. The survival study was terminated at day 120, at which point those mice surviving beyond that time were euthanized for post study analysis. Mice treated with bicarbonate (*p *= 0.03) and DB (*p *= 0.01) had significantly improved survival over untreated and DCA-treated (*p *= 0.2) mice (Figure 4). Three of 15 untreated mice survived after day 120. In the bicarbonate treated group, 8 of 13 mice remained after day 120. Six of 15 mice remained after day 120 in the DCA treated group. In the DB treated group, 10 of 15 mice remained after day 120. The mean survival day was 100, 110, 104, and 112 for untreated, bicarbonate, DCA, and DB treated mice, respectively.

### Analysis of metastases

Metastatic involvement was observed in the intestines, mesentery, lymph nodes, and lungs in all groups (Table 1). All treated mice sustained a lower or equal amount of intestinal, mesentery, and lymph node metastases compared to the untreated group, but no differences were statistically significant. Metastases were present in mice where primary tumors re-occurred after tumor resection and in mice where primary tumors did not re-occur. Overall, there was a significant correlation between tumor re-occurrence and development of metastases (*p *= 0.028). There was no statistical significance between metastases and mice that did not have re-growth of primary tumors. There was no positive correlation between >120 day survival and the presence of metastases (Table 2).

**Table 2:** Metastases observed in mice that survived greater than 120 days

metastases	untreated	bicarbonate	DCA	DB
positive	1	4	3	8
negative	2	3	3	2

**Figure 5:**
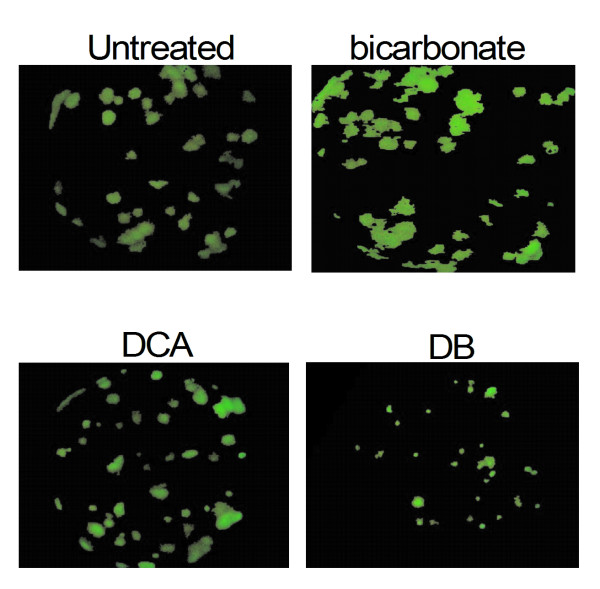
**Representative pulmonary lesions from the four study cohorts**. Green fluorescent lung tumor metastases from necropsies were detected by the Illumatool Bright Light System (LT-9500) using a 470 nm/40 nm excitation filter (Lightools Research). Whole lung images were captured in the frame of view at the same focal plane in the presence of 480-nm excitation and > 490-nm filtered emission.

Most (11 of 14) untreated mice developed pulmonary metastases. Lung metastases were found in 1 of the 11 bicarbonate treated animals. Pulmonary metastases were counted in 6 of 13 DCA treated animals, and 9 out of 15 DB treated mice (Table 1). Average pulmonary lesion sizes in DB treated mice were significantly smaller than lesions from the other groups (*p *< 0.001). The sum area of the pulmonary lesions in DB treated animals was less than half the sum of the lesions in the other groups (Table 3). Representative lung images from each treatment group are presented in Figure 5. The average lesion area in the single bicarbonate treated mouse lung was significantly larger than lesions measured in the untreated mouse lungs (*p *< 0.023) (Table 3). Lung lesion sizes did not correspond with time of death.

**Table 3:** Summary of pulmonary lesions in treatment groups

	Untreated	Bicarbonate	DCA	DB
Lungs analyzed per group	10	1	6	9
Total number of lesions	77	32	71	122
Metastases area sum (lesion pixels)	56090	39289	47428	26897
Metastases area mean (lesion pixels ± SEM)	728 ± 105	1228 ± 220*	668 ± 77	220 ± 18**
Metastases area median (lesion pixels)	447	720	419	160

Metastases lesion areas were determined by measurement of the two-dimensional pixel area. Two diseased lungs, one from the untreated group and one from the DB treated group, could not be reliably measured to include in the analysis. A single asterisk (_*_) designates lesion areas that were significantly different from pulmonary lesions in untreated mice (*p *< 0.05). A double asterisk (_**_) designates lesion areas that were significantly different from pulmonary lesions in untreated mice (*p *< 0.001).

### Cytotoxic and lactate production assays suggest loss of anti-cancer efficacy of DCA in hypoxic tumors

**Figure 6:**
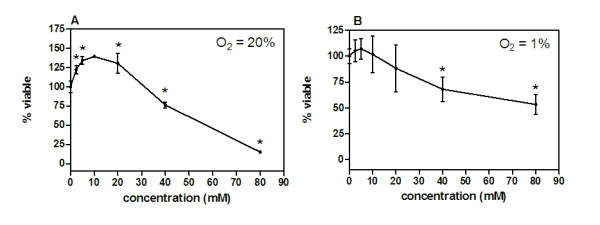
**MDA-MB-231 breast tumor cells were cultured for 24 hours (n ≥ 3) with titrating doses of DCA starting from 80 mM and diluted 2-fold to the lowest concentration of 2.5 mM**. Percent viability for all groups of replicates was normalized by dividing absorbance (590 nm) values by the absorbance values obtained from sham treated (growth media) control cells. Cytotoxicity by DCA in a crystal violet assay was carried out under A) normoxia (O_2 _= 20%) and B) hypoxia (O_2 _= 1%). Asterisks (_*_) designate viable DCA treated cells percentage that were significantly different from 100% viable sham treated cells. Error bars are expressed as SEM.

DCA monotherapy in MDA-MB-231 tumor bearing mice produced no measurable effects against the tumors or metastases. Although previous studies testing lower doses of DCA on tumor growth *in vivo *were effective using other cell lines [17], the tumors in this study were comparatively unresponsive. The effect of titrating doses of DCA under normoxic conditions (O_2 _= 20%) *in vitro *showed that low doses in culture from 2.5 to 20 mM appeared to stimulate cellular viability as demonstrated by a significant 30% increase in viable numbers of cells at these doses after 24 hours. MDA-MB-231 cells exhibited a 25% decrease in viability at 40 mM and an 80% decrease at 80 mM. Viability was 50% at 58 ±5 mM under normoxic conditions (Figure 6A). Similar results were seen after 48 hour incubations with DCA (data not shown).

Microenvironmental conditions such as hypoxia (O_2 _= 1%) can influence drug efficacy against tumor cells. This condition was tested evaluating the cytotoxic effect of DCA on cultured MDA-MB-231 tumor cells. Under hypoxic conditions, DCA doses between 2.5-20 mM had no effect on MDA-MB-231 cell viability. Similar to MDA-MB-231 cells grown under normoxia, a 40 mM DCA concentration significantly reduced viability by 25-30%, but cells were more resistant to increasing concentrations under hypoxic conditions. A DCA dosage of 84 ± 8 mM was required to reduce cell viability to 50% under hypoxia (Figure 6B).

**Figure 7:**
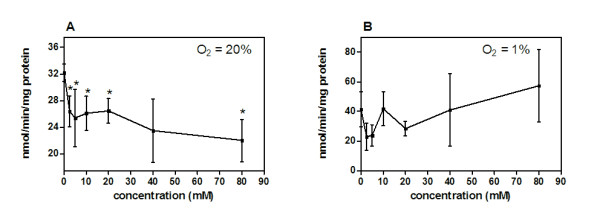
**MDA-MB-231 breast tumor cells were cultured for 24 hours with titrating doses of DCA starting from 80 mM and diluted 2-fold to the lowest concentration of 2.5 mM (n ≥ 3)**. Supernatant lactate was measured as a ratio of the total cellular protein. Lactate production was measured under A) normoxia (O_2 _= 20%) and B) hypoxia (O_2 _= 1%). Asterisks (_*_) designate lactate production in DCA treated cells percentage that were significantly different from untreated control cells. Error bars are expressed as SEM.

We tested DCA in its ability to inhibit glycolysis under normoxia and hypoxia in MDA-MB-231 cells by measuring lactate production. All DCA concentrations from 2.5 to 80 mM inhibited glycolysis between 19% and 29% in MDA-MB-231 cells cultured under normoxia. Reduction in lactate production was significant in all doses (*p *< 0.05) with the exception of the 40 mM concentration (*p *= 0.059) (Figure 7A). Alternatively, under hypoxic conditions, DCA at the same dose range had no measurable effect on lactate production (Figure 7B).

## Discussion

The premise for these experiments was based on mathematical models that suggest the buffering capacity of bicarbonate against tumor acidity could be safely enhanced by addition of DCA (Martin N, Robey I, Gaffney E, Gillies R, Gatenby R, Maini P: Predicting the Safety and Efficacy of Buffer Therapy to Raise Tumor pHe: An Integrative Modeling Study, submitted). The model indicated that inhibition of tumor proton production by a relatively small amount could cause substantial reductions in tumor acidity, without any adverse effects on blood pH. A reagent like DCA fits this criterion because it can reduce extracellular tumor acidity by inhibiting lactic acid production. Moreover, DCA has been shown to selectively target tumor cells [17,18]. The design of this study was to treat our mouse model for breast cancer as similar to a patient study as possible by carrying out a survival experiment with surgical tumor resections.

We confirmed from both urine and serum pH measurements that addition of chronic DCA at the doses administered did not cause significant changes in either urine or serum pH. The urine and serum pH results from DB treated mice were statistically comparable to the measurements in the bicarbonate treated group. We conclude from these findings that DCA does not induce any systemic perturbations that could lead to systemic alkalosis.

Our studies show that none of the treatments reduced primary tumor growth. Our bicarbonate treatment group results were consistent with earlier studies [16]. Other studies have shown that DCA significantly inhibits tumor growth rates *in vivo *[17]. We expected to see this result as well, but found that DCA and DB treated mice had similar primary tumor growth rates to untreated and bicarbonate treated mice. This difference may be explained by the variable effect DCA can have in different tumor cell lines [20,21]. With respect to the metastases results, our findings were similar to a study that administered 5 mM DCA orally to rats injected with a metastatic breast tumor line through the tail vein. There was no measurable effect of the 5 mM dose [21].

It is unknown why DCA treatment did not provide therapeutic benefit against tumors in mice, but the *in vitro *studies may help to explain our findings. The viability studies under normoxic conditions demonstrate that tumor cells have a biphasic response to titrating DCA concentrations. This occurrence is known as hormesis, where low doses can be agonistic and higher doses are toxic to cancer cells [22]. The normoxic *in vitro *viability results suggest DCA concentrations at 20 mM or lower could have a stimulatory effect *in vivo*, though this was not measured. Only at concentrations exceeding 20 mM did DCA reduce viability under normoxia. Under hypoxia, DCA concentrations at 20 mM or lower had no effect on viability. There was a marginal, but significant reduction in viability at DCA concentrations greater than 20 mM under hypoxia. An 80 mM concentration was required to reduce viability 50%.

In the lactate production studies, DCA doses at 20 mM or lower significantly reduced MDA-MB-231 cell glycolytic metabolism under normoxia by approximately 19%. Doses higher than 20 mM inhibited lactate production by 29% under normoxia. No concentration of DCA tested reduced cellular lactate production under hypoxia. The *in vitro *studies suggest; 1) that DCA concentrations greater than 20 mM may have a non-specific effect on MDA-MB231 cells and 2) hypoxia may be a factor contributing to the *in vivo *findings.

MDA-MB-231 mammary xenografts are known to develop regions of hypoxia due to the development of necrotic lesions. Also, unpublished experiments (Robert Gillies laboratory at the University of Arizona) imaging MDA-MB-231 tumors in mice with bioluminescent reporters linked to promoter regions for hypoxia response elements such as VEGF or CAIX demonstrate that tumors develop regions of hypoxia (exhibit bioluminescence) as soon as tumors become palpable (between 100-200 mm^3^). Other studies have shown the link between DCA function and hypoxia. DCA inhibits pyruvate dehydrogenase kinase-1 (PDK1) which inhibits cellular metabolism and causes oxygen consumption resulting in increased hypoxia [23,24].

Although the metastatic load observed in bicarbonate treated mice appeared markedly lower than reported in DB treated mice (especially mesentery and intestinal), the data is insufficient to conclude that bicarbonate monotherapy was more effective at reducing the spread of metastases. The major impact on survival in our cancer model is lung metastases. As seen in the untreated group, most (79%) of the animals showed evidence of metastatic lung lesions. Only one of the bicarbonate treated animals in the analysis was found to have lung metastases, but there were two (of the four) spontaneous deaths that occurred in this group and one in the untreated group where we were unable to analyze the lungs. Nonetheless, a two-tailed Fischer's exact analysis shows that including the bicarbonate treated group spontaneous deaths as lung metastases positive (23%) and the untreated as negative for lung metastases, frequency of lung metastases would still be significantly lower in the bicarbonate treated mouse group (*p *= 0.02). It was surprising that the percentage of DB treated mice with lung metastases was 60%, and not significantly different from the occurrence of lung metastases in the untreated mouse group, indicating the bicarbonate monotherapy as a more favorable treatment modality at least in this tumor model. The findings suggest a potential risk in the DB treatment that would need to be investigated further in other models for cancer.

Analysis of the pulmonary lesions revealed that DB treatment resulted in significantly smaller mean tumor metastases compared to all other groups. The mean lesion diameter in DB treated mouse lungs was no larger than 30% of the lesion diameters in the other treatment groups. In a previous study it was reported that smaller tumor metastases in lungs was correlated significantly with bicarbonate treatment alone, but this was a 30 day experiment using β-galactosidase expressing MDA-MB-231 cells and the mean tumor diameters were about 87% of the lesion diameters measured in the untreated group [16]. The comparison of tumor lesion sizes between the metastasized lungs in all the study groups appears to suggest that DB treatment inhibits the growth of these metastases (Table 3). However, given the prevalence of non-metastatic lungs in the bicarbonate treated group, it could be argued that DCA has more of a stimulatory effect, especially if O_2 _concentrations are more normoxic, and may even compete with bicarbonate therapy. This conclusion is supported by the *in vitro *findings which show that lower concentrations of DCA increase cell viability under normoxia.

Improved survival was a significant outcome in all groups treated with bicarbonate. There seemed to be little measurable therapeutic effect in DCA treated mice, suggesting bicarbonate was a driving component in the survival study. The results are consistent with earlier findings [16], but the mechanisms are not well understood. More than half the primary tumors in bicarbonate and DB treated mice did not return after surgical resections. We attribute the lower frequency of primary tumor re-occurrence to the improved survival rates in these groups. The difference between bicarbonate treated groups (bicarbonate and DB) and non-bicarbonate treated groups (untreated and DCA) in tumor re-occurrence after surgeries is statistically significant (*p *< 0.05). It may be possible that the effect of systemic bicarbonate could potentiate the wound healing process after surgical disruption of the tumor region. The role of bicarbonate ions (HCO_3_^-^) in wound repair was first reported in a study investigating gastric mucosal repair in cats systemically administered sodium bicarbonate. It was concluded that systemic excess of HCO_3_^- ^facilitated superficial mucosal repair [25]. The molecular mechanisms driving this effect are unknown, but other reports have shown that blocking the Na^+^-HCO_3_^- ^co-transport (NBC) with isothiocyanate (ITC) inhibits epithelial restitution, the process of epithelial migration involved in wound healing. Additionally, cell migration during restitution is dependent on glycolysis for energy [26]. This is notable because NBC functionally cooperates with monocarboxylate lactate transporter (MCT-1), a glycolysis-associated enzyme. Extracellular HCO_3_^- ^increases NBC activity, which in turn enhances MCT activity leading to an increase in intracellular pH [27], a physiological condition conducive to glucose metabolism [28]. It is thought that the upregulated glycolytic activity of tumor cells offers a competitive advantage over surrounding host cells [14]. The epithelial wound healing processes after tumor resection in conjunction with an increase in extracellular bicarbonate may improve the competitive state of some host cells in the tumor microenvironment, and result in prevention of primary tumor re-growth. Although further evidence for these processes is required, it suggests that an optimal time to administer systemic bicarbonate would be after a tumor surgery.

## Conclusions

This study confirms earlier reports about the role of systemic bicarbonate in the inhibition of metastatic spread from primary tumors, but highlights the limitations of this approach. While DCA has been shown both safe and effective against tumor cells in other studies, the findings reported here concur with the most recent investigations warning of potential pitfalls with DCA use [23,24]. First, it is not universally effective against all cancer cells. Secondly, tumor hypoxia serves as a confounding micro-environmental factor against DCA efficacy. The unpredictable effect of DCA against tumors therefore signals caution against using this agent as a therapeutic approach until new studies can determine the molecular role of DCA in different tumor microenvironments.

## Abbreviations

DCA: dichloroacetate; DB: DCA-bicarbonate; PDH: pyruvate dehydrogenase; PDK: pyruvate dehydrogenase kinase; IACUC: Institutional Animal Care and Use Committee; SCID: severe combined immunodeficient; EMSS: Experimental Mouse Shared Services; GFP: green fluorescent protein; NBC: sodium bicarbonate co-transport; ITC: isothiocyanate; MCT-1: monocarboxylate lactate transporter.

## Competing interests

The authors declare that they have no competing interests.

## Authors' contributions

IR performed all experiments. IR and NM performed the statistical analysis. IR drafted the manuscript and NM revised the manuscript. IR and NM were involved in conception and design of the study and participated in the discussion and interpretation of the results. All authors read and approved the final manuscript.

## Pre-publication history

The pre-publication history for this paper can be accessed here:

http://www.biomedcentral.com/1471-2407/11/235/prepub
